# Emergence of cutaneous leishmaniasis in Nepal

**DOI:** 10.1186/s41182-021-00359-3

**Published:** 2021-09-09

**Authors:** Kishor Pandey, Anup Bastola, Gong Haiyan, Uttam Raj Pyakurel, Basu Dev Pandey, Shyam Prakash Dumre

**Affiliations:** 1 Central Department of Zoology, Tribhuvan University, Kathmandu, Nepal; 2 Sukraraj Tropical and Infectious Diseases Hospital, Kathmandu, Nepal; 3 Shanghai Veterinary Research Institute, Chinese Academy of Agricultural Sciences, Shanghai, China; 4 Epidemiology and Diseases Control Division, Kathmandu, Nepal; 5 Central Department of Microbiology, Tribhuvan University, Kathmandu, Nepal

**Keywords:** Cutaneous leishmaniasis, Emergence, Elimination, Nepal, Visceral leishmaniasis

## Abstract

**Background:**

Cutaneous leishmaniasis (CL) is endemic in 70 countries worldwide. Nepal is considered non-endemic for CL and hence the control program is targeted to visceral leishmaniasis (VL) only. Here, we report the emergence of CL cases in different parts of Nepal.

**Methods:**

We analyzed the CL and VL cases reported to Epidemiology and Diseases Control Division (EDCD), Ministry of Health and Population, Nepal through District Health Information System 2 (DHIS-2) and Early Warning and Reporting System (EWRS) during the past 4 years (2016–2019). Any laboratory-confirmed case was included in the study. Demographic and clinical details of each patient were transcribed into Excel sheets, verified with the case report forms and analyzed.

**Results:**

VL has been reported in Nepal since 1980, but CL was reported very recently. From 2016 to 2019, 42 CL cases were reported from 26 different hospitals to EDCD which had been diagnosed on the basis of clinical presentation, and laboratory findings (demonstration of amastigotes in Giemsa-stained smears and rK39 test results). Majority of the patients (31.0%, 13/42) visited to the hospital within 1–6 months of onset of lesions. Facial region (38.1%, 16/42) was the common place where lesions were found ompared to other exposed parts of the body. CL was successfully treated with miltefosine for 28 days. The majority of CL patients did not have history of travel outside the endemic areas and there was no report of sandfly from these areas.

**Conclusion:**

These evidences highlight that the Government of Nepal need to pay more efforts on CL and include it in differential diagnosis by clinicians, and plan for an active surveillance when the country is targeting leishmaniasis elimination by the year 2025 with the decreasing number of VL cases.

**Supplementary Information:**

The online version contains supplementary material available at 10.1186/s41182-021-00359-3.

## Background

Leishmaniasis is caused by protozoan parasite of genus *Leishmania* which is transmitted from one person to others by the bite of an infected female phlebotomine sandfly. Infection with *Leishmania* species results into one of the three different clinical forms, namely cutaneous leishmaniasis (CL), mucocutaneous leishmaniasis, and visceral leishmaniasis (VL) (the most serious form) [1]. Among these, CL is the most common form of leishmaniasis and endemic in 70 countries with majority of cases reported from Afghanistan, Algeria, Brazil, Pakistan, Peru, Saudi Arabia, Syria, and currently expanding to newer locations including the Indian subcontinent [2, 3]. It is estimated that between 600,000 and 1 million new cases of CL occur worldwide annually [1]. The clinical features of CL include varying number of skin ulcers/lesions on exposed parts of the body, leaving life-long scars and serious disability or stigma or nodular lymphangitis [4].

Nepal is one of the five endemic countries for VL globally [5]. Most of the elimination efforts are being focused on VL-endemic areas of Nepal [6]. Nepal is a landlocked country bordering India in the South, East and West; and China in the North. The country is divided into seven provinces [Province 1, Province 2 (no specific names have been designated yet to two provinces), Bagmati, Gandaki, Lumbini, Karnali and Sudurpaschim], 77 districts and three ecological regions namely terai (southern plains), mid hills and northern mountains which ranges from 70 to 8848 m above the sea level. Over half of the populace lives in the terai districts of Nepal, where vector-borne diseases namely, malaria, dengue, Japanese encephalitis, and kala-azar are endemic [7–11].

There are several challenges that need to be addressed effectively, before VL elimination becomes a reality; for example, treatment failures, incidence of co-infections, post-kala-azar dermal leishmaniasis (PKDL), the expansion of VL into new areas and more importantly the recent reports of sporadic CL cases in different areas of Nepal [12]. As of now, very little is known about the emergence of CL and its persistence in Nepal. According to recent national guidelines on kala-azar elimination, 2019, the districts of Nepal have been classified into ‘endemic’, ‘endemic doubtful’ and ‘non-endemic’. Endemic districts are those districts where full cycle of transmission has been demonstrated at any given time and at least one locally acquired case in the last 10 years. Endemic doubtful districts are those where full cycle of transmission has never been demonstrated, but at least one locally acquired case in the last 10 years or full cycle of transmission has been demonstrated at any given time, but no case has been reported in the last 10 years (0 case or no data). Non-endemic districts are those where full cycle of transmission has not been demonstrated and no locally acquired case has been reported in the last 10 years, but locally acquired case had been reported earlier. There are 18, 50, and 9 districts designated as VL-endemic, endemic doubtful and non-endemic districts, respectively. VL elimination and control activities in Nepal have been focused in 18 endemic districts [6]. While VL has been endemic in the southern plains (terai) of Nepal for many years now [7, 13, 14], a small number of imported CL cases has also been reported [15]. The first CL case was reported in Nepal in the year 1998 followed by a few intermittent case reports (mostly imported) [15–19]. There is no solid evidence of autochthonous transmission of CL in Nepal.

CL does not cause mortality; however, the nature of lesions and infection is commonly occurring in exposed parts (face, limbs, waist) which increase distress and disfigurement among CL cases. Therefore, early and correct diagnosis of CL is very important for the elimination of leishmaniasis from this region. In this report, we describe the recent emergence of CL cases in Nepal and its potential implication in the leishmaniasis elimination in the country and region. The aim of the present study is to carry out epidemiological studies of CL in current changing scenario.

## Methodology

The present study was a retrospective study where we collected secondary data of leishmaniasis from Epidemiology and Disease Control Division (EDCD), Department of Health Services, Ministry of Health and Population, Nepal. CL cases with no travel history had been reported only very recently in Nepal.

**Fig. 1:**
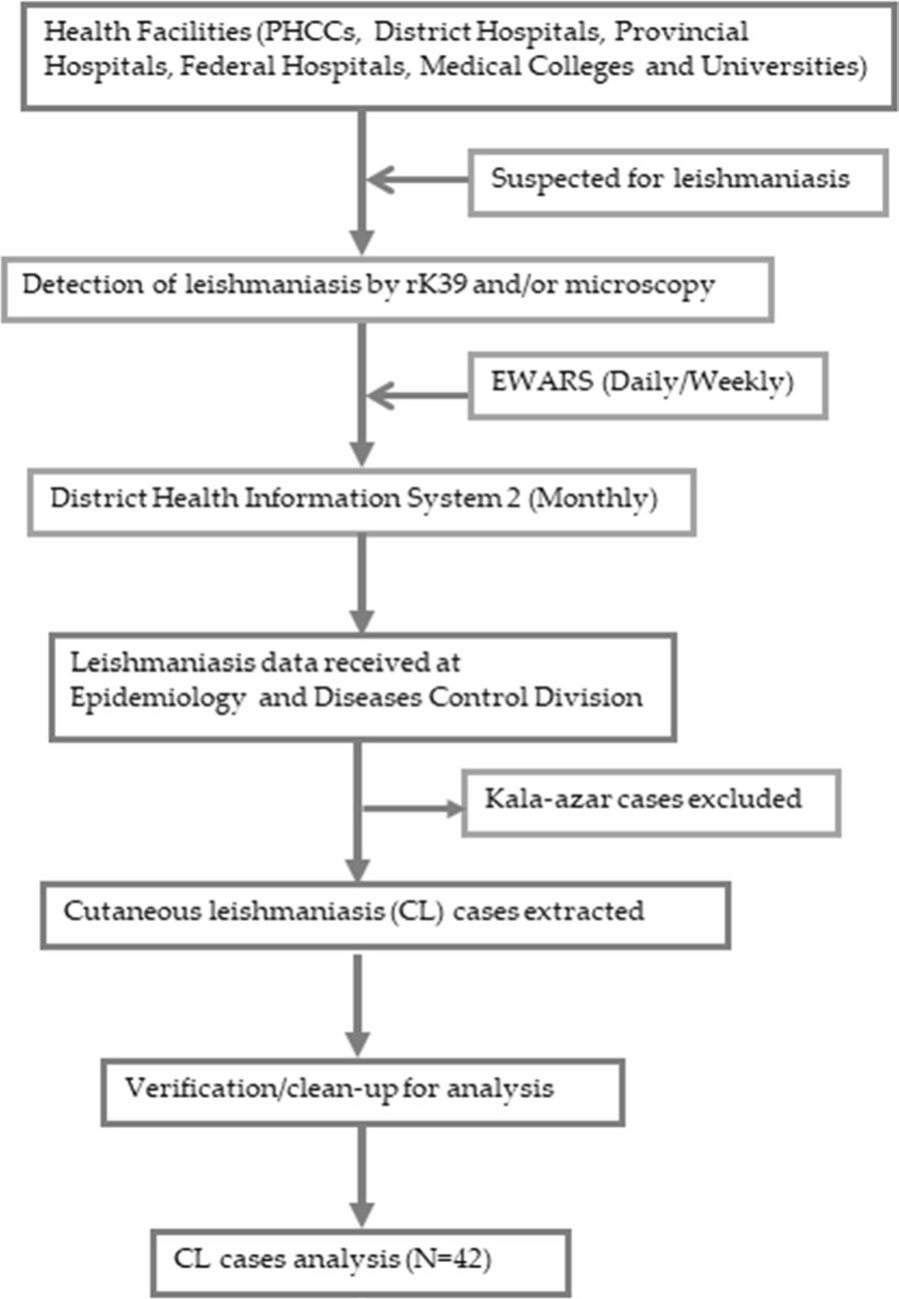
Outline of the leishmaniasis data collection process in Nepal. *PHCC* Primary Health Care Center, *CL* cutaneous leishmaniasis, *EWARS* Early Warning and Reporting System

Kala-azar (KA) or VL surveillance program in Nepal had been using an online platform called District Health Information System 2 (DHIS-2) and Early Warning and Reporting System (EWARS). During the duration covered in the present study, all the leishmaniasis suspected patients were diagnosed at Primary Health Care Centers (PHCCs: below district level) and treatment was done beyond the district and higher level (District Hospitals, Provincial Hospitals, Federal Hospitals, Medical Colleges and Universities) throughout the country. Health care providers at these facilities are trained for the leishmaniasis case definitions, case identification, referral as required and reporting. Each health facility prepares KA treatment registration information which has to be recorded and reported. KA treatment register should include all confirmed leishmaniasis data that had been diagnosed and treated. Past history of VL was asked and PKDL cases were excluded in the present study. Daily/weekly, the health facility reported the leishmaniasis cases to EWARS (90% kala-azar cases have been reported through EWARS) and end of the month all data complied to DHIS-2 through online portal system from treatment center and send to EDCD (Fig. 1). The potential duplication of leishmaniasis cases were checked on a monthly basis by comparing key identification information. EDCD is the national authority to manage leishmaniasis elimination program that uses data from national surveillance system. Based on these data, EDCD prepares control and elimination strategies for KA. CL cases reporting also includes same online platform that used for KA.

The present study included all patients who attended and reported with lesions and clinically suspected of CL from January 2016 to December 2019. Each patient’s demographic data (age, sex, and area of residence) and clinical details (site and duration of the lesion, and treatment response) were duly obtained from EDCD. Data included in the patient’s case report forms were transcribed into MS Excel sheets for verification and analysis. We performed the descriptive analysis of the data and different variables were presented as percentages and frequencies as appropriate. We also mapped the CL cases in the country’s geo-political maps to reflect the geographic (provincial and district-wise) distribution and endemicity of CL (endemic, endemic doubtful and non-endemic) in Nepal.

## Results

**Fig. 2:**
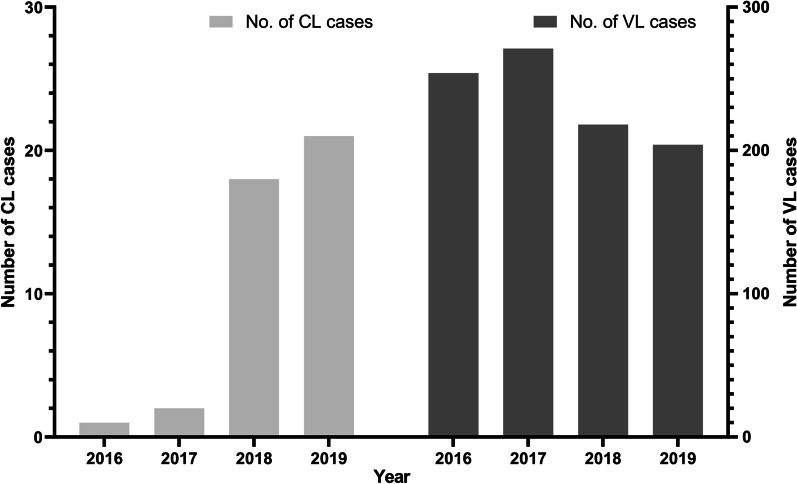
Year wise number of cutaneous leishmaniasis (CL) and visceral leishmaniasis (VL) cases in Nepal, 2016–2019

To understand the trends of leishmaniasis in the last 4 years (2016–2019) in Nepal, we plotted the number of CL and VL cases. The trends of CL and VL cases during this period were clearly different. There was increase in CL cases but the number of VL cases was decreasing in the national scenario (Fig. 2). The number of CL cases was smaller and reported since 2016.

**Fig. 3:**
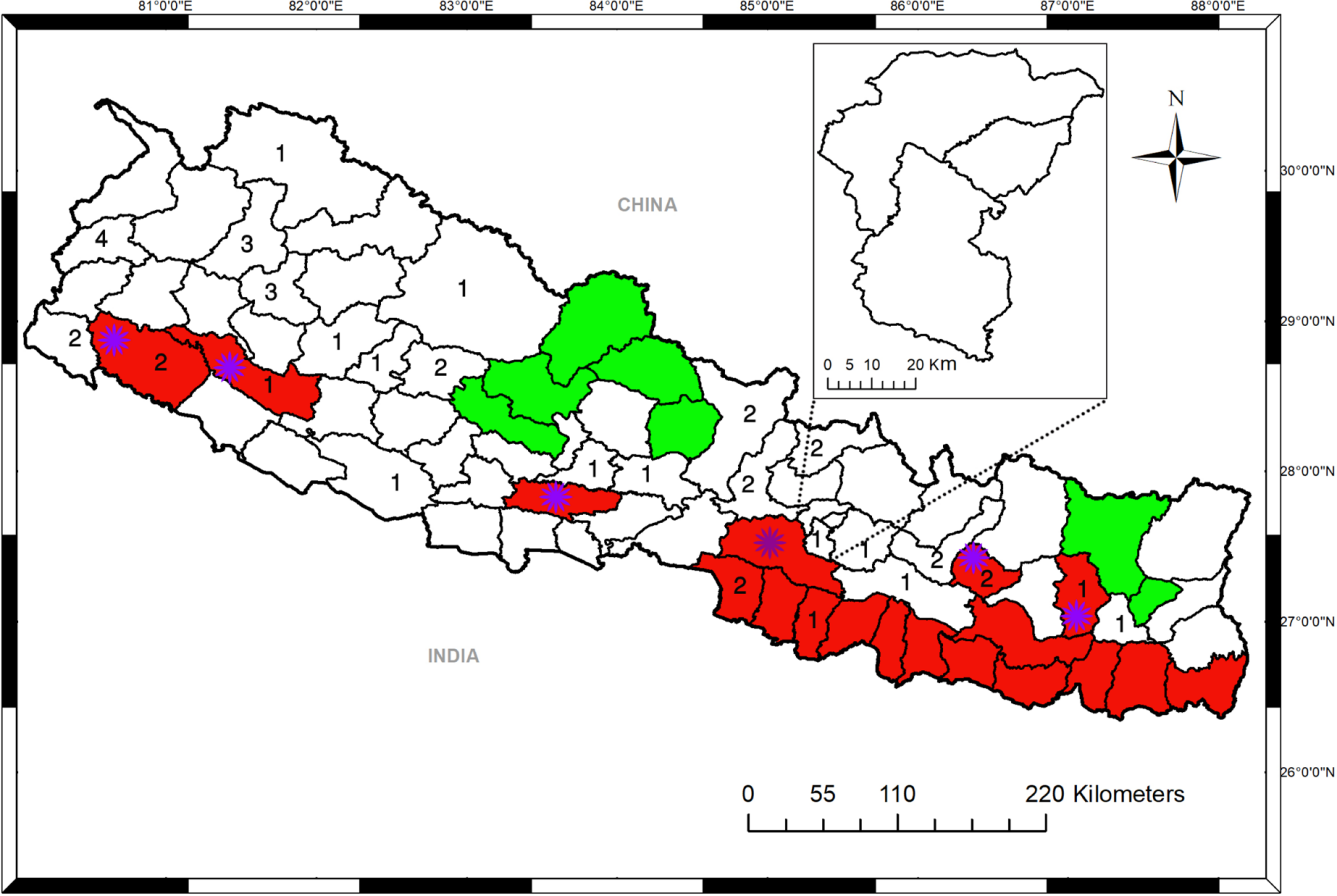
Map of Nepal showing visceral leishmaniasis (VL) endemic, endemic doubtful and non-districts districts with reported CL cases. Red color shows the VL-endemic districts (*n* = 18), green color shows non-endemic districts (*n* = 9) while the rest are endemic doubtful districts (*n* = 50). Numbers indicate CL cases distribution by district (*n* = 26) in the present study. Asterisk shows newly added endemic districts (*n* = 6)

A total of 42 clinically diagnosed patients with CL were reported to different hospitals from 27 districts, during January 2016 to December 2019 (Fig. 3). Of these, 66.7% (28/42) were males and 33.3% (14/42) were females. These cases were in the age range of 9–85 years, average age being 39 years (Table 1).

**Table 1:** Demographic findings of cutaneous leishmaniasis patients in Nepal, 2016–2019

Variable	Category	Percentage of cases, % (*Number of cases/Total cases*)
Age (years)	< 20	26.2 (11/42)
	21–40	26.2 (11/42)
	> 40	47.6 (20/42)
Sex	Male	66.7 (28/42)
	Female	33.3 (14/42)
Geography	Terai	19 (8/42)
	Hill	64.3 (27/42)
	Mountain	16.7 (7/42)
Districts	Endemic	21.4 (9/42)
	Endemic doubtful	78.6 (33/42)
Travel history	Yes	19 (8/42)
	No	81 (34/42)

Most of the CL cases (31.0%, 13/42) presented to the hospital within 1–6 months of onset of lesions (self-noticed) while 21.4% (9/42) and 9.5% (4/42) patients presented to the hospital between 7 and 12 months and after one year of symptom onset (visible lesions), respectively (Table 2). Lesions were found on the exposed areas of the body, mainly on the facial region (38.1%, 16/42) and upper limbs (19.0%, 8/42). The trunk and lower limb involvement were seen in 4.8% (2/42) of each CL patients. 47.6% (20/42) patients had single lesion while 19.1 (8/42) cases had multiple lesions in their body.

**Table 2:** Clinical findings of cutaneous leishmaniasis, 2016–2019

Variable	Category	Percentage of cases, % (*Number of cases/Total cases*)
No. of lesions	Single	47.6 (20/42)
	Multiple	19.1 (8/42)
	Data not available	33.3 (14/42)
Site of lesions	Face and neck	38.1 (16/42)
	Hands	19.0 (8/42)
	Legs	4.8 (2/42)
	Trunk	4.8 (2/42)
	Data not available	33.3 (14/42)
Interval between onset of lesion to hospital visit	< 1 month	0
	1–6 months	31.0 (13/42)
	7–12 months	21.4 (9/42)
	> 1 years	9.5 (4/42)
	Data not available	38.1 (16/42)
Treatment	Miltefosine	59.5 (25/42)
	Liposomal Amphotericin B	7.1 (3/42)
	Data not available	33.3 (14/42)
Diagnosis	Microscopic	83.3 (35/42)
	rK39	16.7 (7/42)

Giemsa-stained skin smears showed the presence of amastigote (LD bodies) in 83.3% (35/42) cases while the rest 16.7% (12/28) patients were rK39 positive. All the patients were treated with miltefosine for 28 days and a few cases were also treated with liposomal amphotericin B. All the patients responded to the treatment and recovered.

Of the 42 CL cases, 81% (34/42) were from non-endemic areas, whereas 19% (8/42) cases were from endemic areas (Table 1, Fig. 3). The vast majority of the CL cases 78.6% (33/42) had no history of travel to a known VL-endemic area(s), while 21.4% (9/42) had travel history to VL-endemic districts in Nepal or countries like India and United Arab Emirate (UAE). Geographically, 64.3% (27/42), 16.7% (7/42) and 19.0% (8/42) patients were, respectively, from hilly, mountainous and terai regions of Nepal (Table 1, Fig. 3).

**Fig. 4:**
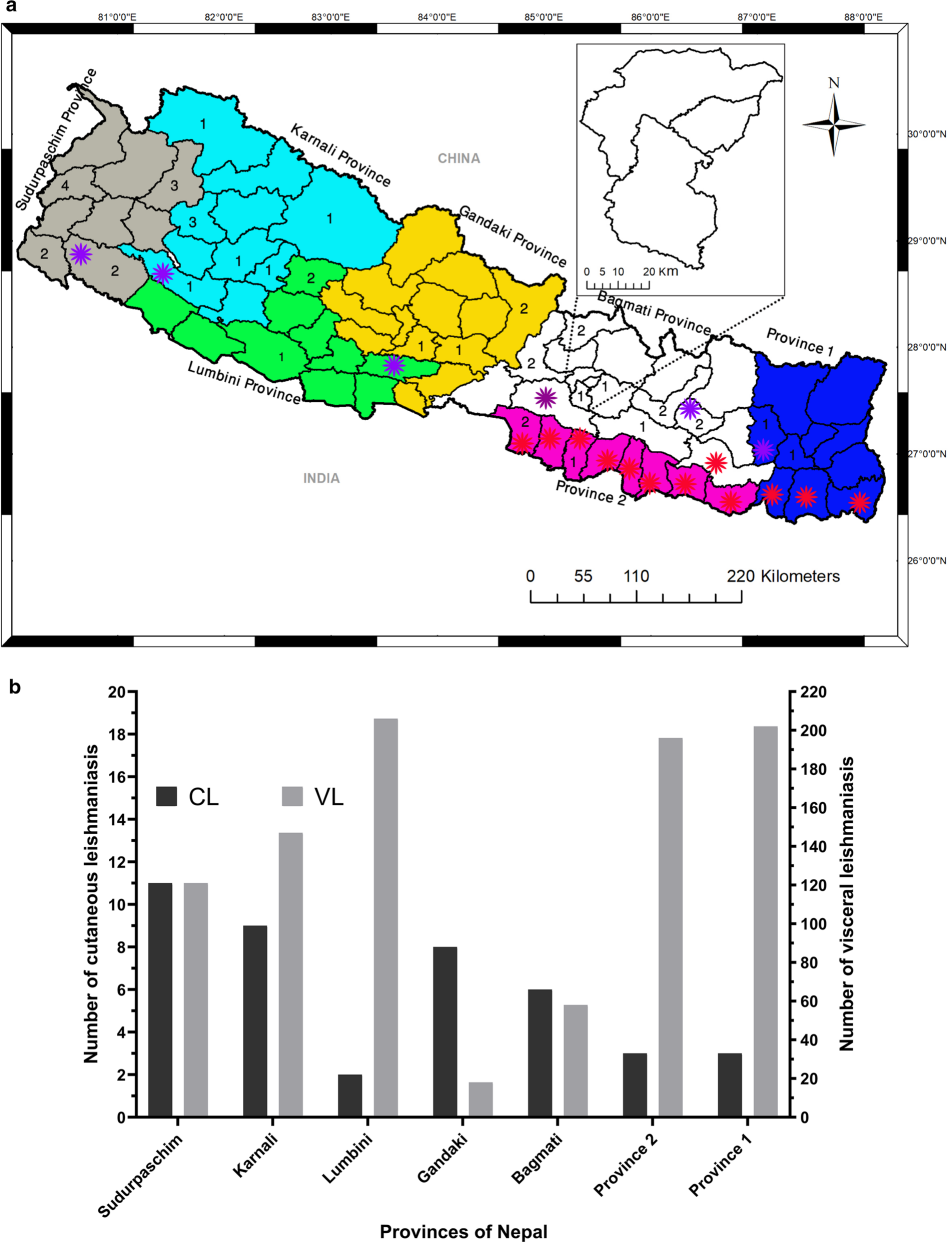
Trend of leishmaniasis cases reported during 2016-2019 in Nepal. **a** Map of Nepal showing provinces of Nepal with different colors. Red and purple asterisks show 12 endemic and recently added 6 endemic districts for VL in Nepal. **b** The graph shows provincial breakdown of cutaneous leishmaniasis (CL) and visceral leishmaniasis (VL) cases in Nepal

The first case of VL was reported in 1960s in Nepal. The control program initially identified 12 VL-endemic districts from the Terai (southern plains) region of Province 2 (8 districts) and Province 1 (4 districts). In 2016, six new districts—mostly from hilly region (2 districts from province 1 and 1 district each from Bagmati, Lumbini, Karnali and Sudurpaschim provinces) were added to the list of endemic districts (Fig. 4a). The newly added 6 districts confirmed the local transmission of VL supported by epidemiological and entomological evidences. The overall trends of VL incidence decreased with the introduction of elimination plan in 2005 (Additional file 1: Figure S1). However, VL cases were expanding from ‘endemic areas’ (Provinces 1 and 2) to ‘endemic doubtful areas’ (Lumbini, Karnali and Sudurpaschim provinces) (Fig. 4b). Both VL and CL had been expanding towards the newer geographic locations in Nepal. Interestingly, the higher number of CL cases were found in Sudurpaschim (*n* = 11) and Karnali (*n* = 9) provinces, respectively, followed by Gandaki (*n* = 8) and Bagmati (*n* = 6) provinces (Fig. 4b).

## Discussion

To our knowledge, this is probably the first instance that a bulk number of CL cases found in Nepal which used to be very rare incidence in the past [15, 16, 18–21]. In the present study, the highest number of CL cases were from the age group > 40 years similar to previous reports [15, 16]. In contrast, a recent report from India also found a higher incidence in 10–19 years age group [22]. CL was observed most commonly in males compared to females (male:female = 2:1). In Nepal, males are mostly involved in outdoor works where there is higher chances of sandfly bites [15].

Most of the patients had single lesion in the exposed parts of the body (face, neck and limbs). These findings suggest that the exposed body parts were more accessible to vectors and sandfly easily bites in those areas. Similar results were also reported in other studies conducted in Srilanka and Nepal [15, 23].

CL is a dermal manifestation commonly caused by various species of *Leishmania* that is transmitted by female sandfly. Our study provides important information about emergence of CL cases in VL-endemic-doubtful and non-endemic districts of Nepal in addition to the endemic districts (Table 1). Earlier, a few CL cases with unclear travel history had also been reported in Nepal [15, 21]. These evidences are indicative of an established CL transmission in Nepal. Therefore, it is utmost important for concerned authorities to take rapid actions to achieve timely elimination targets before it becomes a worse epidemic. Most physicians in Nepal do not consider CL in the differential diagnosis, perhaps due to the exclusive presence of VL in the past [24]. Moreover, CL shows similar clinical features like other skin disease (such as leprosy which is still prevalent in Nepal) resulting in substantial delays in appropriate treatment due to misdiagnosis. Physicians should be made aware of the recent emergence of CL and its diagnosis in Nepal, even though CL is rarely fatal [15, 21]. It causes considerable morbidity with widespread involvement, disfigurement, and scary look and potential societal stigma, apart from challenging the leishmaniasis elimination goals.

CL is the most common type of leishmaniasis with worldwide distribution and it has re-emerged in several new areas globally [1, 2]. After the first CL cases in 1998, only few CL cases have been reported till now in Nepal [15, 17, 19, 20], and majority of them had a travel history to CL endemic countries. In the present study, CL cases were also observed in the mountains and hilly districts of Nepal. Those districts are considered endemic doubtful and non-endemic districts for VL in Nepal [6]. VL has been the major public health problems in Nepal since the first case identified in 1953 by an Indian Scientist Raghavan (EDCD, 2004). The government focused in the 18 VL-endemic districts from terai region of Nepal. As of 2020, sporadic VL cases have been reported from 59 of 77 districts in Nepal [6]. Despite the expansion of VL cases to many new districts, overall incidence has been gradually declining. However the cases of CL have been increasing in both endemic doubtful and non-endemic districts of Nepal [25], similar to what we observed.

In 2005, the three south Asian countries (Nepal, India and Bangladesh) launched an initiative to eliminate VL from the region, aiming to reduce the disease incidence to ≤ 1 VL case per 10,000 population per year at the district level. To achieve this target, EDCD/Government of Nepal formulated VL elimination plan in 2005 which was revised as the National strategic guidelines on VL elimination in 2010. It was further updated in 2014 to introduce liposomal amphotericin B and combination therapy in the national treatment protocol. In 2019, National guidelines on kala-azar elimination was revised to strengthen the surveillance as well as treatment approaches with single dose of liposomal amphotericin B. The national kala-azar elimination program focuses mainly on early diagnosis and complete treatment, improved case management, effective disease and vector surveillance, social mobilization and partnership, and integrated vector control.

Until recently, VL was thought to be the major clinical form of *Leishmania* infection in Nepal. When we plotted the number of CL and VL cases reported in Nepal, we found that both diseases are expanding from endemic districts (Province 2) to endemic doubtful districts (Karnali and far-western provinces) (Fig. 3). An increasing number of VL cases from areas previously considered non-endemic, and growing CL cases in Nepal are among the major challenges in an effort to eliminate the disease [11, 26]. Majority of the previously reported CL cases in Nepal were those staying aboard where CL was endemic (India and UAE) or had been reported and returned back to Nepal [16, 18, 19]. In contrast, the CL patients in the present study did not live in the VL program districts of Nepal.

Previously, majority of CL cases had a history of travel to endemic countries. But our results showed most of the CL patients did not have history of overseas travel. A large number of Nepalese migrant workers are employed in gulf countries (0.33, 0.28 and 0.29 million per year in 2016, 2017 and 2018, respectively) [27]. There is possibility of getting CL infection in these countries. When they return home, they might bring infections and perhaps establish a local transmission since there is abundance of sandfly vectors in different parts of Nepal. This could be a possible reason for local transmission and increased the CL cases in recent years. After the implementation of VL elimination plan/program in Nepal from 2005, total number of VL cases have significantly decreased with a recently changing epidemiological trend. VL cases from endemic districts from eastern *Terai* region have now expand to western *Terai* and hilly districts of Nepal.

Presently, the exact reasons for CL emergence, that too in both endemic doubtful and non-endemic districts, in Nepal is unclear. Two hypotheses may explain the emergence of CL. First, the vector of leishmaniasis (sandfly) might be spreading to new areas of Nepal. Climate change is found to be correlated with shifting of vector’s geographic range worldwide [28]. This could be a possible explanation for vector expansion and leishmaniasis transmission beyond the existing endemic areas. We previously reported expansion of VL in both endemic-doubtful and non-endemic districts of Nepal [11, 26]. Second, although CL is caused by different *Leishmania* species, it might also be a plausible explanation that VL causing *Leishmania* species could have caused CL in Nepal. *L. donovani* is the causative agent for VL, but a recent study indicated that it may cause CL too [21]]. This hypothesis is also supported by the distribution pattern of VL and CL in 2019 in Nepal where CL cases were mostly found in the VL reported areas (Fig. 3). These emerging CL cannot be simply overlooked, though more research needs to be carried out to confirm the potential sources of CL, including the molecular profiling of *Leishmania* species in both patients and vectors from CL reported areas. Moreover, it would be of interest to determine whether the vector of leishmaniasis has adapted to hilly environments (higher altitudes), enabling the disease to spread to this area.

### Limitation

We included the CL cases from EDCD data which received data base from national surveillance system. This study stands as descriptive study with limited number of samples. Furthermore, in the current leishmaniasis surveillance/reporting system, only limited information is included (age, sex, limited clinical information, and laboratory diagnosis, treatment and treatment outcomes), and often many values are missing. Due to lack of other variables and precise data, we could not analyze the risk factors for CL. In the current reporting system, all the data/ information related to confirmed leishmaniasis cases are required to report, while the data on suspected cases are not reported. This has made it difficult to make proper inferential statistics without the suspected or leishmaniasis negative data (only positive data are reported and recorded in the health information management system for leishmaniasis). PCR test could not be performed to identify the *Leishmania* species that caused CL. Owing to this, the possibility for selection bias could not be completely ruled out. As we built up the very first base-line information (data) in the present study, we will certainly work on a more robust prospective study with much detailed variables/parameters included to identify the risk/predictive factors of CL/ CL emergence in Nepal which would help in the elimination activities. We believe that through this base-line information, we will be able to recommend the control and elimination program on specific improvements (additional information) in the online surveillance system based on the existing scenarios and recommend further operational research on CL in the country.

## Conclusions

This study has highlighted the issue of an important neglected disease and it is expected that it will draw the attention of the policy makers to formulate protocol for the effective surveillance, management and control of leishmaniasis. If CL cases are increased, it will adversely impact the leishmaniasis elimination in the Indian subcontinent due to open borders shared with Nepal. Such a growing number of CL cases from a country embarking for leishmaniasis elimination is certainly a regional concern too. We, therefore, highly recommend that future research and control program efforts should focus not only on VL and endemic districts, but also on CL and endemic doubtful and non-endemic districts to achieve the elimination goals.

## Supplementary Information

**Additional file 1: Figure S1.** Trends of VL cases from 2005 to 2019 in Nepal.

## Data Availability

Not applicable.
