# Supplementary figures and images for: Emergence of cutaneous leishmaniasis in Nepal
Source: Trop Med Health. 2021 Sep 9;49:72. doi: 10.1186/s41182-021-00359-3 (PMC8428101; doi:10.1186/s41182-021-00359-3)

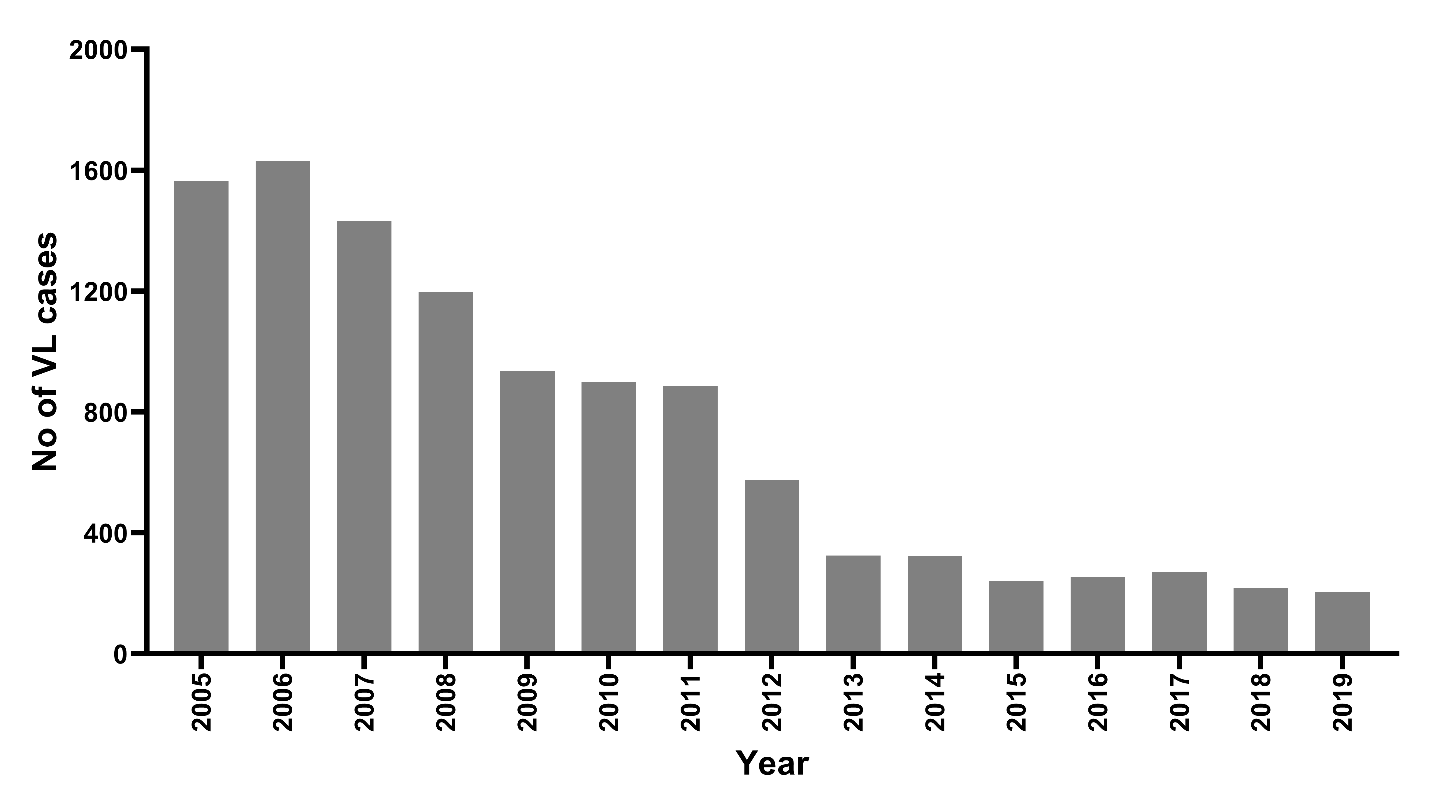


Figure S1. Trends of VL cases from 2005-2019 in Nepal.

Supplement: Supplementary file 1 — Additional file 1: Figure S1. Trends of VL cases from 2005 to 2019 in Nepal. [file 41182_2021_359_MOESM1_ESM.docx]
